# Successful Pregnancies During Crizotinib Therapy for Anaplastic Lymphoma Kinase Positive Lymphoma

**DOI:** 10.1002/hon.70038

**Published:** 2025-01-14

**Authors:** Veronica Guglielmana, Francesco Maria Fusi, Valentina Giardini, Federica Cocito, Carlo Gambacorti‐Passerini

**Affiliations:** ^1^ Department of Medicine and Surgery University of Milano‐Bicocca Monza Italy; ^2^ Hematology Division and Bone Marrow Unit IRCCS San Gerardo dei Tintori Monza Italy; ^3^ Department of Maternal Fetal and Pediatric Medicine ASST Papa Giovanni XXIII Bergamo Italy; ^4^ Obstetrics Unit Department of Maternal Fetal Medicine IRCCS San Gerardo dei Tintori Monza Italy

**Keywords:** aggressive non‐Hodgkin's lymphoma, lymphoproliferative disorders, pregnancy, quality of life

## Abstract

Anaplastic lymphoma kinase positive (ALK+) anaplastic large cell lymphoma (ALCL) typically affects young individuals and, despite high responsiveness to cytotoxic drugs, relapses occur in over 50% of patients. Crizotinib has improved outcomes, but its management in patients desiring parenthood remains an issue. This study presents the first description of four successful pregnancies during crizotinib treatment for ALK+ALCL: a female patient achieving two pregnancies through assisted reproductive technologies (ART), temporarily discontinuing crizotinib and maintaining a complete remission (CR), and a male patient conceiving naturally while on continuous therapy. These cases demonstrate crizotinib potential to support conception without compromising disease control, even in the absence of specific guidelines on treatment discontinuation.

## 1 Introduction

ALCL accounts approximately for 2% of all non‐Hodgkin lymphomas and, among the adult nodal T‐cell lymphomas, it represents the third most common subtype [1]. WHO‐HEAM5 recognizes three different entities: ALK+, ALK‐ and Breast implant‐associated lymphoma [2]. Systemic ALK+ALCL is most frequently observed in the first three decades of life with a slight male predominance. ALK+ALCLs are defined by genetic alterations of the ALK gene, causing the abnormal expression of ALK protein. The most frequent genetic aberration involved is the t (2; 5) (p23; q35) chromosome translocation, that leads to the fusion of the 3′ part of the ALK gene on chromosome 2 with the nucleophosmin (NPM) gene on chromosome 5. Despite the high responsivity to cytotoxic drugs, for example, cyclophosphamide‐doxorubicin‐vincristine, etoposide‐prednisone (CHOEP) and Brentuximab vedotin containing regimens, relapses develop in > 50% of patients; relapsed/refractory (R/R) disease bears a poor outcome, thus representing a major concern [3]. The availability of crizotinib, an inhibitor of ALK, ROS proto‐oncogene 1 (ROS1), and Mesenchymal‐Epithelial Transition (MET) receptor tyrosine kinases changed this dismal situation. Crizotinib produces long‐lasting responses in 2/3 of R/R ALK+ALCLs [4, 5, 6, 7, 8]. However, some clinical unmet needs remain. The management of crizotinib therapy in patients who have a desire of parenthood is one of such concern.

Here we provide the first description of four cases of successful pregnancies during crizotinib treatment for ALK+lymphomas.

## 2 Results

Patient 1, presented with ALK+ALCL stage IIIB (supraclavicular, axillary, paratracheal, hilar, pericephalopancreatic and iliac lymph nodes involvement) when she was 30 years old. Treatment with VACOP‐B (etoposide‐doxorubicin‐cyclophosphamide‐vincristine‐prednisolone‐bleomycin) combination chemotherapy produced CR. Eight years later, at the age of 38, relapse occurred with nodal and hepatic involvement, along with positive t (2; 5)/NPM1‐ALK signal by real‐time polymerase chain reaction (RT‐PCR) on peripheral blood (PB). A second CR was obtained with eight courses of the antibody‐drug conjugate Brentuximab vedotin, followed by autologous stem cells transplant (ASCT) with myeloablative conditioning lomustine‐etoposide‐cytarabine‐melphalan (CEAM) regimen. At day +47 post ABMT, B symptoms and inguinal adenopathies reappeared. At this point, crizotinib monotherapy was started at 250 mg twice daily with good tolerance and low‐grade side effects, such as fluid retention. CR, both metabolic and molecular, was reached after two months of therapy. The latter was evaluated through qualitative RT‐PCR assay for NPM1‐ALK transcript on PB, whereas the metabolic response was assessed by total body fluorodeoxyglucose‐positron emission tomography/computerized tomography (FDG‐PET/CT). After two years of continuous treatment and sustained remission, at the age of 41, the patient expressed a desire for motherhood using ART as she experienced iatrogenic menopause following ASCT; ovarian stimulation and oocytes cryopreservation were conducted before it. The pregnancy was attained through in vitro fertilization (IVF) after warming of four frozen oocytes; a double embryo transfer (ET) was performed with cryopreservation of two blastocysts. Crizotinib was interrupted two weeks before ET. The first‐trimester scan confirmed a single gestational sac. Down's syndrome screening (combined test and non‐invasive prenatal test ‐ NIPT) yielded negative results, while the screening for preeclampsia indicated a potential heightened risk; low‐dose aspirin (LDA) was administered from 13 weeks until 36 weeks. Ultrasound scans showed regular fetal growth without abnormalities. The pregnancy was uneventful, except for a pauci‐symptomatic SARS‐CoV‐2 infection at the 36th week for which she received a prophylactic treatment with enoxaparin sodium. NPM1‐ALK RT‐PCR, assessed monthly, remained negative throughout the pregnancy. Spontaneous rupture of membranes occurred at 39th week. After 24 h, labor was induced with oral misoprostol and syntocinon infusion, resulting in vaginal delivery of a healthy male newborn weighting 3300 g, appropriate for gestational age, 44° percentile according to INeS (Italian Neonatal Study) chart, with APGAR scores of 9 and 9 at 1 and 5 min, respectively. The postpartum period was uncomplicated. She opted not to breastfeed. Two weeks after birth, crizotinib was resumed at 250 mg daily. The child is now 27‐month‐old, in excellent general conditions with normal cognitive and motor development for his age. After maintaining a stable molecular response for 20 months on a continuous regimen of crizotinib, the patient, now 43 years old, expressed her desire for a second pregnancy, given the availability of two cryopreserved blastocysts. Treatment with crizotinib was interrupted again. The first ET failed, while the second one, attempted 4 weeks later, was successful. Cardiac activity was detected in a single gestational sac after 5 weeks. At the 16th week, the pregnancy was progressing normally, although a small decidual detachment (14 × 5 mm) was detected. To support placental health, LDA was initiated, similar to the approach taken in the previous pregnancy. Delivery occurred via cesarean section at 35+4 weeks gestation, caused by spontaneous preterm rupture of membranes. The male neonate had a birth weight of 2760 g, an APGAR score of 9 and 10 at 1 and 5 min, respectively, and an umbilical arterial pH of 7.42. The postpartum course was uneventful, with no observed complications. NPM1‐ALK RT‐PCR, monitored monthly throughout the pregnancy, remains negative.

Patient 2, a 25‐year‐old male affected by ALK+ALCL stage III_S_ (left axillary and supraclavicular nodes and focal splenic involvement) and treated with six courses of CHOEP. At the conclusion of chemotherapy, total body FDG‐PET/CT showed a CR, but NPM1/ALK RT‐PCR on PB remained positive. Considering the high risk of relapse with the persistence of the t (2; 5) signal after the end of chemotherapy [9], crizotinib 250 mg twice daily was started, achieving molecular response within one month. No adverse events of any grade were reported. The patient conceived a child after four years of continuous therapy and in stable remission, despite our recommendation to use barrier methods of contraception. No interruption of crizotinib was made. Five years later, he fathered a second child, at the age of 34. Both babies were female with normal weight at birth (respectively 3430 and 3500 g) and no complications occurred during their pregnancies. At present, the children are 7 and 2 years old, and both in good health.

## 3 Discussion/Conclusions

Crizotinib is a highly specific and not mutagenic drug active in both adult and pediatric ALK+lymphomas [10]; however, ALK is selectively expressed in the central nervous system; in addition, the MET gene, the other target of crizotinib, can also produce Non‐Syndromic Autosomal Recessive Deafness when germinally mutated [11].

Understanding the placental permeability of crizotinib is critical due to its implications for potential fetal exposure during pregnancy. Ex vivo human placental perfusion studies have revealed that crizotinib significantly accumulates in placental tissue, with the capacity to inhibit key placental transporters and impair trophoblast cell viability [12]. To further assess fetal exposure, researchers have developed physiologically‐based pharmacokinetic (PBPK) models. These models integrate ex vivo perfusion data with in vivo conditions, providing a robust framework to simulate and predict the extent of fetal exposure to crizotinib during pregnancy [13]. However, clinical data on crizotinib use in pregnancy remain limited and in our two cases crizotinib was interrupted before conception.

Some case reports of pregnancies in women undergoing crizotinib therapy for ALK+lung adenocarcinoma are present in the literature. However, in most of these cases, the pregnancy was already ongoing at the time of the cancer diagnosis, and treatment with crizotinib was initiated either during the pregnancy or after it [14, 15]. Moreover, no reported cases of men who conceived while undergoing crizotinib therapy exist, regardless of the underlying disease.

Due to these considerations, we advised the first patient to interrupt the drug during both pregnancies, while the second patient did not discontinue crizotinib when he conceived his two daughters. Since no data exist regarding the duration of crizotinib treatment in ALK+ALCL patients and the possibility of discontinuing treatment during remission, it is encouraging to observe that Patient 1, who discontinued crizotinib on two occasions—the first after 40 months of therapy for a period of 10 months, and the second, two years later, also for 10 months—did not show any signs of lymphoma regrowth during these interruptions. Patient 2 course indicates that concurrent treatment with crizotinib possibly does not affect spermiogenesis.

These cases do not pretend to provide definitive answers regarding the discontinuation of crizotinib or the safety of pregnancy during treatment; prospective studies and pharmacodynamic investigations will be required to fully address these questions. Our aim is to signal the feasibility of conceiving during crizotinib treatment considering not only the control of the underlying disease, but also the importance of quality of life and the patient's needs, including the desire for parenthood.

## Author Contributions

**Veronica Guglielmana:** conceptualization, investigation, writing–original draft preparation. **Francesco Maria Fusi:** investigation. **Valentina Giardini:** investigation. **Federica Cocito:** investigation, writing–reviewing and editing. **Carlo Gambacorti‐Passerini:** conceptualization, investigation, writing–reviewing and editing.

## Consent

The authors certify that informed consent was obtained from the patients for the publication of all images, clinical data and other data included in the manuscript.

## Conflicts of Interest

The authors declare no conflicts of interest.

### Peer Review

The peer review history for this article is available at https://www.webofscience.com/api/gateway/wos/peer-review/10.1002/hon.70038.

## Data Availability

No data associated with our study has been deposited into a publicly available repository. The data that has been used is confidential.
